# Weaving community-based participatory research and co-design to improve opioid use treatments and services for youth, caregivers, and service providers

**DOI:** 10.1371/journal.pone.0297532

**Published:** 2024-04-18

**Authors:** Roxanne Turuba, Christina Katan, Kirsten Marchand, Chantal Brasset, Alayna Ewert, Corinne Tallon, Jill Fairbank, Steve Mathias, Skye Barbic

**Affiliations:** 1 Foundry, Vancouver, British Columbia, Canada; 2 Providence Health Care, Vancouver, British Columbia, Canada; 3 Faculty of Medicine, University of British Columbia, Vancouver, British Columbia, Canada; 4 Department of Occupational Science and Occupational Therapy, University of British Columbia, Vancouver, British Columbia, Canada; 5 Canadian Centre on Substance Use and Addiction, Ottawa, Ontario, Canada; 6 Centre for Advancing Health Outcomes, Vancouver, British Columbia, Canada; 7 Foundry Victoria, Victoria, British Columbia, Canada; 8 Providence Research, Vancouver, British Columbia, Canada; Universidad Internacional de La Rioja, SPAIN

## Abstract

Integrating the voices of service users and providers in the design and delivery of health services increases the acceptability, relevance, and effectiveness of services. Such efforts are particularly important for youth opioid use treatments and services, which have failed to consider the unique needs of youth and families. Applying community-based participatory research (CBPR) and co-design can facilitate this process by contextualizing service user experiences at individual and community levels and supporting the collaborative design of innovative solutions for improving care. However, few studies demonstrate how to effectively integrate these methods and engage underserved populations in co-design. As such, this manuscript describes how our team wove CBPR and co-design methods to develop solutions for improving youth opioid use treatments and services in Canada. As per CBPR methods, national, provincial, and community partnerships were established to inform and support the project’s activities. These partnerships were integral for recruiting service users (i.e., youth and caregivers) and service providers to co-design prototypes and support local testing and implementation. Co-design methods enabled understanding of the needs and experiences of youth, caregivers, and service providers, resulting in meaningful community-specific innovations. We used several engagement methods during the co-design process, including regular working group meetings, small group discussions, individual interviews and consultations, and feedback grids. Challenges involved the time commitment and resources needed for co-design, which were exacerbated by the COVID-19 pandemic and limited our ability to engage a diverse sample of youth and caregivers in the process. Strengths of the study included youth and caregiver involvement in the co-design process, which centered around their lived experiences; the therapeutic aspect of the process for participants; and the development of innovations that were accepted by design partners.

## Introduction

There are growing efforts to involve service users (i.e., patients and families) and service providers in the design and delivery of health services to improve quality of care [1]. While service providers make important recommendations based on their knowledge of the system and experiences delivering care, patients and families hold unique insights on the appropriateness of services based on their circumstances and preferences [2]. Involving patients and families has led to improvements in service delivery [1], reductions in hospital admissions [3], the development of family-oriented programs [4], and advances in models of care [5] and organizational processes [6]. Patient and family engagement also has the potential to change the organizational culture within healthcare settings and promote collaboration among patients, families, and service providers [7–9].

One approach to patient and family engagement in healthcare is to use co-design, which engages service users, health care providers, and designers as equal partners to design innovative and effective solutions to improve quality of care [10]. Co-design and other closely related design approaches (e.g., user-centered design, human-centered design (HCD), design thinking) have been increasingly applied in healthcare to improve patient and family experiences and outcomes [11–17]. Although these design approaches all center around understanding end-users’ experiences and involve some form of engagement in the design process, co-design specifically engages end-users as *active* design partners, rather than design participants (e.g., informants, testers, sources of information) [18]. This acknowledges patients’ and families’ expertise in their own lived experience and gives them decision-making power in the design process, while allowing them to dictate their level and mode(s) of engagement. Community-based participatory research (CBPR) shares similar principles of reciprocity and collaboration as co-design by actively engaging community members in the research process to define the problem, design the study, and translate the results into action [19]. These methods are rooted in social activism and have been increasingly used in health research to address health disparities among underserved populations [20–23].

Applying CBPR and co-design can help researchers and healthcare providers bridge the gap between research and practice by improving their understanding of service user experiences and needs within the healthcare system and developing patient-centered innovations that are acceptable and responsive to local contexts. These approaches may improve the experiences and outcomes of community-based opioid use treatments/services for youth, as current organizational- and system-level policies often hinder service providers’ ability to meet the individual needs of youth, which are closely related to their local context [24]. For instance, British Columbia (BC) guidelines on opioid agonist treatment (OAT) best practices require people to access a pharmacy daily to acquire their OAT, which does not consider the barriers of living in rural and remote communities (e.g., transportation, travel distance, pharmacy hours) [24]. Although CBRP and co-design methods can limit the generalizability of findings to a specific community or region, these investigations are crucial for developing solutions that work in practice.

Improving youths’ access to opioid use treatments/services is particularly important given the ongoing opioid toxicity crisis in Canada, which accounted for 32,632 deaths between January 2016 and December 2022 [25]. Youth (ages 15–24) represent 21% of these deaths and have the fastest growing rates of opioid-related toxicity hospitalizations [26]. Using substances early in life can have detrimental effects on youths’ substance use trajectories and is predictive of developing health and psychosocial problems later in life [27–29]. Current guidelines to treat opioid use disorder (OUD) among youth include a combination of pharmacological (e.g., OAT) and psychosocial (e.g., cognitive behavioral therapy) interventions, as well as family-oriented treatments and long-term recovery services [30]. Yet, youth experience a number of barriers when trying to access these services, including age-based restrictions [31, 32], service provider hesitancy to prescribe OAT to youth [33, 34], a lack of confidentiality and privacy [32, 35], and limited youth-friendly service options [32, 34, 36, 37], which may explain why youth are less likely to access or remain engaged in opioid use treatments/services compared to adults [38–41]. Several qualitative studies have found that youth often feel unsafe accessing adult-oriented harm reduction services (e.g., safe consumption sites, OAT), which can prevent them from accessing life-saving programs [32, 34, 42].

Further, youths’ treatment preferences and goals often do not align with the way services are currently delivered [34, 43–46]. For example, studies exploring youth perspectives on OAT demonstrate that youth often view OAT as a short-term solution to manage opioid cravings while addressing other concerns that play a role in their opioid use (e.g., basic needs, mental health, relationships) rather than a long-term treatment option as often applied in adult populations [34, 39, 46]. Although the engagement of people who use drugs in the design and delivery of opioid use treatments/services is increasing, youth and families are often still excluded in these efforts [36, 44]. It is therefore imperative that we better understand and respond to the unique treatment needs of youth who use opioids and the families who support them.

Although CBPR and co-design are increasingly being applied to improve healthcare experiences, few studies report how to integrate and apply these methods and how to effectively engage underserved populations in the co-design process [47–50]. No other studies to date describe the use of co-design methods to improve youth opioid use treatments/services. As such, the objective of this paper is to describe how the methodological processes and procedures from CBPR and co-design can be woven together to improve community-based opioid use treatments/services for youth, caregivers, and service providers. Using two case study examples from the Improving Treatment Together (ITT) Project, this paper addresses an important methodological gap for other health service researchers considering these two methods. Further, the case studies demonstrate how these methods can be applied to co-develop health service innovations in the context of substance use treatment for priority populations, such as youth [35, 44, 51].

## Methods

### Study design and setting

**Fig 1:**
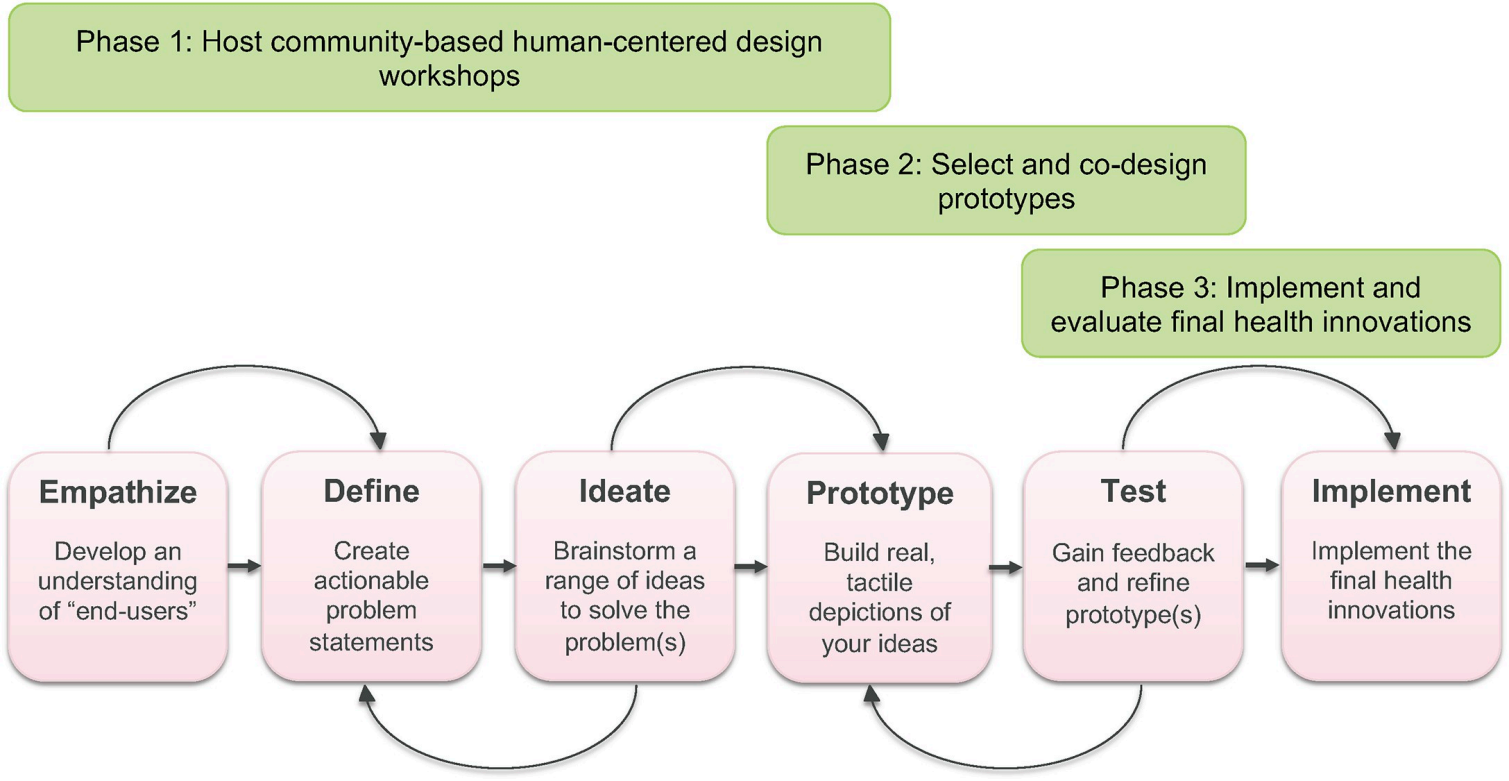
ITT project phases using CBPR and co-design methods.

The ITT project combined CBPR and co-design methods to improve the experiences and outcomes of 1) youth accessing community-based opioid use treatments/services, 2) their caregivers, and 3) their services providers in four communities across BC, Canada (Kelowna, Prince George, Victoria, and Vancouver). The project used design thinking, a human-centered methodology that follows six iterative steps (see Fig 1) [52] to collect evidence regarding the current state of youth opioid use treatments/services (Phase 1) and co-design (Phase 2), implement, and evaluate (Phase 3) innovative solutions that are directly informed by stakeholders who access or deliver these services.

The findings from Phase 1 have been published elsewhere [24, 53, 54]. Briefly, Phase 1 began by developing a partnership between the Canadian Centre on Substance Use and Addiction (CCSA), a national organization providing leadership on addressing substance use related harms, and Foundry Central Office, a central hub for a provincial network of integrated youth services in BC. Community partnerships were established with four Foundry centers, which provide mental health, substance use support, physical and sexual health, peer support, and social services to youth ages 12–24. Four youth team members with lived/living experience of opioid use from each partnering community were onboarded to the project and trained in CBPR, design thinking, and group facilitation. Harmonized ethics approval was received by the University of British Columbia/Providence Health Care (Study ID: H19-02077). Youth, caregivers, and service providers were recruited between September 29^th^, 2019, and February 3^rd^, 2020, to take part in workshops in their community. Four workshops were held in November 2019 (Kelowna and Prince George) and five workshops in February 2020 (Vancouver and Victoria) that explored participants’ experiences and needs when accessing/providing opioid use treatments/services (Empathize) and created meaningful and actionable problem statements based on these needs (Define). Participants were then asked to identify a wide range of ideas to address these needs (Ideate) and create low-fidelity prototypes by defining what the solution would entail, how it would be implemented, who would be involved, and its intended impact (Prototype). All participants provided written consent at the beginning of the workshop.

Phase 2 focused on translating the Phase 1 findings into action by developing three of the prototypes with our community partners. The ITT project team used an internal decision-making framework (see Additional File 2 in Marchand et al., 2021 [55]) to determine which of the 31 low-fidelity prototypes from Phase 1 were most feasible to develop and implement within the scope of the ITT project and the context of the COVID-19 pandemic. These prototypes involved a wide range of solutions, including education programs; interdisciplinary care teams; resources to improve system navigation, support caregivers, and create youth-friendly spaces; and new programs to improve youths’ access to services, housing, financial support, and meaningful connections. The framework assessed the affordability, sustainability, and time commitment needed to develop each prototype. It also considered whether the prototype was identified by multiple communities and stakeholder groups and whether it could be scalable to other communities and populations. This resulted in a condensed list of prototypes, which was shared with community partners (e.g., executive directors, center managers, implementation managers, youth team members). Partners were asked to assess each prototype’s potential for local impact, novelty, organizational match, and the timeframe needed for successful co-design. While project resourcing would be provided for this phase, partners also considered community resources (e.g., staff time, existing initiatives that could complement/support the co-design process) in selecting a prototype that would have the utmost impact in the community. Ultimately, the ITT project team selected three prototypes for co-design that would address the needs of all three priority stakeholder groups (youth, caregivers, and service providers). Community champions (CCs) from each partnering organization were hired to support local development with a co-design team of youth, caregivers, and/or service providers from their community. Community partners, CCs, and the co-design teams were also involved in informing the implementation and evaluation of the final products in Phase 3. For a full description of the project protocol, please refer to Marchand et al., 2021 [54].

In this paper, we will focus on describing how our team wove CBPR and co-design methods in Phase 2 of the project (see Table 1) by presenting two of the prototypes chosen for development and implementation as case study examples. These solutions include a caregiver handbook and a virtual OAT guide, both of which can be found at https://foundrybc.ca/ittproject/.

**Table 1:** CBPR and co-design methods used during ITT project Phase 2.

Phase 2: Select and co-design prototypes	
CBPR methods	Co-design methods
Select appropriate prototypes to co-design with community partners and youth team members	Co-design prototypes with youth, caregivers, or service providers during the Phase 1 workshops
Hire CCs from partnering organization to lead the local development and implementation of each prototype and support recruitment of co-design partners	Hire CCs with lived experience to lead the local development and implementation of each prototype and support recruitment of co-design partners.
CCs help identify co-design partners and informants to participate in the co-design process	Recruit youth, caregivers, and service providers to participate in the co-design process
Work with CCs to determine initial engagement methods	Determine future engagement methods with co-design partners
Project team members and CCs co-facilitate interviews, working group meetings, and small group discussions with co-design partners and informants	CCs with lived experience co-facilitate interviews, working group meetings, and small group discussions with co-design partners and informants
CCs help identify local contractors to co-produce the final innovations	Co-design teams help design the final innovations
Community partners review the innovations	Test innovations with youth, caregivers, and/or service providers who were not involved in the co-design process

CC: Community Champion

### Case study 1: Co-designing a handbook to support caregivers of youth with substance use disorders in Victoria, BC

#### Rationale for prototype selection

This case study reports the co-design of a caregiver handbook with community partners at Foundry Victoria. The original idea came from a caregiver workshop in Victoria, BC (Phase 1) and was chosen in response to the community’s need for more tools to help caregivers support youth who use opioids. It also offered an opportunity to support caregivers who may not be comfortable engaging with others in a group setting. The handbook was designed to address some of the barriers described by caregivers in the Phase 1 workshops when trying to navigate the system for their young person, such as limited capacity, long wait times, age-based treatment policies, and a lack of connection across services and sectors [54]. These barriers were compounded by the lack of knowledge caregivers had about OUD and the associated harms, as well as the emotional rollercoaster caregivers experienced trying to keep their young person alive. As such, this handbook aimed to provide caregivers with relevant information about substance use and substance use disorders and how to navigate the healthcare system and relevant situations, while also providing emotional support through storytelling.

#### Co-design team

**Fig 2:**
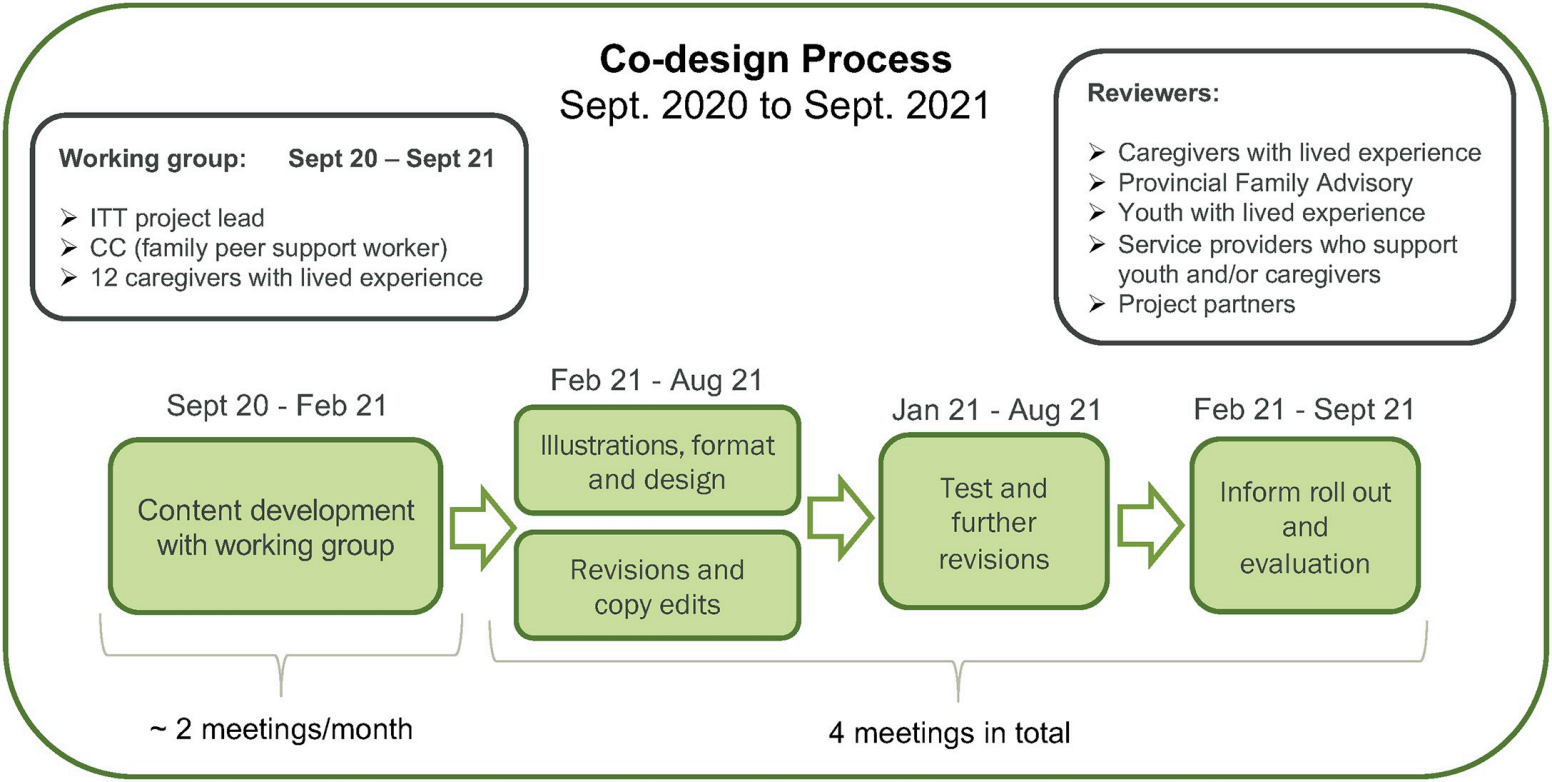
Co-design process for the caregiver handbook.

A family peer supporter was hired as a CC (author CB) to co-lead the local development and implementation of the handbook with an ITT project team lead (author RT). The CC recruited caregivers from two family peer support groups in Victoria who had previously been invited to the Phase 1 workshops. Twelve caregivers who had experience parenting a youth with OUD joined the Phase 2 co-design team as members of a working group that met regularly with the project team lead and CC to develop and review the handbook’s content, illustrations, format, and design (see Fig 2). Four caregivers attended one working meeting before disengaging due to various reasons (i.e., lack of capacity, technology barriers, opioid-related toxicity events). The CC had lived experience parenting a youth with OUD and therefore played a large role in the content development and provided caregivers with social and emotional support during the co-design sessions (and thereafter). All participating caregivers were provided with a $25/hour honorarium for their time.

#### Co-design process

The content development for the caregiver handbook took place from September 2020 to February 2021 in bi-weekly meetings held virtually (via Zoom) due to the COVID-19 pandemic (see Fig 2). Each virtual meeting was audio-recorded, with permission from the group, to ensure the content retained the caregivers’ narrative. An ITT project team member also took notes and captured impactful quotes throughout the meetings. The first meeting was used to introduce the project and co-design team members and develop terms of reference, including defining the group’s roles and responsibilities, timelines, honoraria, and a community agreement, which was referred to at the start of every meeting to create a safe space to share experiences. The co-leads (CC and ITT project team lead) then shared an environmental scan of existing resources that were similar to the prototype from the Phase 1 workshop and asked caregivers to share their vision for the handbook and brainstorm potential topics. Caregivers highlighted the importance of personal stories to help others feel less alone in their journey and to underscore that recovery is not linear and looks different for everyone in order to reduce stigma and shame. They also hoped the handbook would provide other caregivers with relevant resources and encourage them to reach out for support.

Ten main topics were identified, which evolved throughout the co-design process. Caregivers were informed that the total number of topics included in the final version of the handbook would depend on the progress rate to meet project deadlines and budget restrictions. They were asked to prioritize sections based on what they were most passionate or knowledgeable about and believed to be most important to include, which were the focus of subsequent meetings. Break-out rooms of 3–4 caregivers were facilitated by 1–2 ITT project team members, who shared their screen(s) and took notes as caregivers conversed. Prompting questions were used to help guide the development of the chapters, including “*What was your experience/journey like*?*”*, *“What were some of the barriers*?*”*, *“What did help*?*”*, *and “What do you wish you had known*?*”*. The written content was refined by the ITT project team lead and revised with the working group to ensure it reflected their ideas and experiences. Caregivers were also given the opportunity to write sections outside of meeting hours and share personal stories. Additional research and consultations with content experts in youth substance use took place to incorporate subject-specific evidence.

The handbook content went through multiple rounds of revisions by the project team, working group, and a copy editor. As per CBPR methods, the handbook was reviewed by the communications and leadership teams from all partnering organizations (Foundry Victoria, Foundry Central Office, and CCSA). The handbook was then tested with service providers and caregivers in community to acquire external feedback. This included Foundry’s Provincial Family Advisory, which was comprised of 29 family members with lived experience representing all nine Foundry centers operating in communities across the province at the time of the project, and additional caregivers from Victoria who were invited to participate in Phase 2 but declined to take part. Feedback on language/readability, flow/order, consistency/clarity, the overall content, and whether the handbook met its intended outcomes was collected using a feedback grid. Only two caregivers responded with the feedback form, while others provided feedback directly via email or with edits and comments directly in the handbook document. Service provider comments were particularly helpful in refining the list of resources and information on navigating different services. An ITT youth team member with lived experience of opioid use also revised the handbook to ensure that the information provided to caregivers was relevant to their experiences and did not cause any unintentional harms to youth.

The handbook’s artistic process was also informed by the caregiver working group. We hired a vendor specializing in graphic facilitation, recording, and illustration in October 2020 to create illustrations for the handbook and develop its layout and design (i.e., color pallets, fonts, and page size). Ideas were discussed with the caregiver working group and the designer and three rounds of revisions were conducted with the working group to ensure the final product met their vision.

The working group also informed the implementation and evaluation of the handbook. A twofold approach to implementation was proposed by caregivers: 1) an online, freely available version of the handbook to be hosted on the Foundry website, and 2) distribution of hard copies to local organizations that serve caregivers and youth. The second approach was supported by the local CC and working group members. To inform the evaluation outcome measures, the evaluation lead (author AE) joined a working group meeting to acquire the outcomes caregivers anticipated the handbook would have. The online version of the handbook can be found on the Foundry website (foundrybc.ca).

#### Co-design process evaluation

Anonymous midpoint and endpoint surveys were distributed to working group members via email by the evaluation lead, who was independent of the co-design team, to evaluate the process. The surveys were adapted from the Public and Patient Engagement Evaluation Tool (PPEET) [56] and used Likert-type and open-ended questions where caregivers could elaborate on their responses and provide further feedback. Surveys were completed in Qualtrics^®^ and did not collect any personally identifiable information. The midpoint survey was distributed two months into the development process, while the endpoint survey was distributed six months into the process, once the handbook content was finalized.

The survey questions and findings are presented in Table 2. The eight caregivers who remained involved throughout the co-design process completed the surveys. Overall, caregivers described feeling adequately supported to participate in the co-design meetings and expressed feeling satisfied with their role. Caregivers felt they were able to express their views freely and that a wide range of views were shared and discussed. Caregivers reported the chosen innovation was reflective of the needs of their community and anticipated that it would have a positive impact. Although most caregivers felt they learned valuable information and skills during the co-design process, not all caregivers felt hopeful about the future of opioid use treatment services for youth as a result of the work.

**Table 2:** Caregiver handbook midpoint and endpoint process evaluation survey results.

Midpoint and Endpoint Survey Questions	Midpoint survey (n = 8) ^a^	Endpoint survey (n = 8) ^a^
	Mean ± SD (Median)	Mean ± SD (Median)
**Part A: Communication and support for participation**		
I have a clear understanding of the purpose of the working group	4.38 ± 1.41 (5)	4.33 ± 1.63 (5)
The supports I need to participate are available (i.e., prep materials, online devices/software, compensation, etc.)	4.63 ± 0.52 (5)	4 ± 1.55 (4.5)
The COVID-19 pandemic made it more challenging for me to participate in this group ^c^		3 ± 1.10 (3)
I have enough information to contribute to the topic being discussed	4.75 ± 0.46 (5)	4.67 ± 0.52 (5)
Meetings are scheduled at times that are convenient to me	4.5 ± 0.53 (4.5)	4 ± 0.63 (4)
My role and responsibilities were clearly explained to me when I joined the working group	4.75 ± 0.46 (5)	4 ± 0.63 (4)
My role within the working group meets my expectations	4.5 ± 0.53 (4.5)	4.33 ± 0.52 (4)
I have learned valuable information/skills during my time as a member of the working group	4 ± 0.53 (4)	4.33 ± 0.82 (4.5)
**Part B: Sharing your views and perspectives**		
I am able to express my views freely	5 ± 0 (5)	4.5 ± 0.84 (5)
I feel that my views are heard	4.88 ± 0.35 (5)	4.5 ± 0.84 (5)
A wide range of views are shared and discussed	4.63 ± 0.52 (5)	4.5 ± 0.84 (5)
The individuals participating in the working group represent a broad range of perspectives on the topic of opioid use treatment services for youth	4 ± 0.93 (4)	4.33 ± 0.52 (4)
**Part C: Impact and influence of the working group**		
As a result of my participation in the working group, I anticipate I will be/I am better informed about opioid use treatment services for youth in communities in BC	4.13 ± 0.64 (4)	4.33 ± 0.52 (4)
As a result of my participation in the working group, I anticipate I will have/I have increased capacity to make informed decisions about youth opioid use treatment services in communities in BC	4.13 ± 0.99 (4)	4.17 ± 0.75 (4)
I am confident the input provided throughout this working group will be used by the ITT project team and their project partners (i.e., Foundry)	4.25 ± 0.46 (4)	4.67 ± 0.52 (5)
I think that the prototype we have chosen responds to the needs identified by members of my community	4.38 ± 0.52 (4)	4.67 ± 0.52 (5)
I anticipate that the prototype will achieve its intended objectives	4.38 ± 0.52 (4)	4.83 ± 0.41 (5)
I anticipate that the prototype will have a positive impact on opioid use treatment services for youth in communities in BC ^b^	4.63 ± 0.46 (4)	3.83 ± 1.17 (4)
**Please indicate the degree to which you think the prototype will achieve the following specific objectives**: ^c^		
I think this resource will be useful to parents and caregivers		5 ± 0 (5)
I think parents and caregivers will be satisfied with this resource		4.67 ± 0.52 (5)
I would share this resource with other parents and caregivers		5 ± 0 (5)
I think parents and caregivers will have an increased awareness of opioid use treatment services after accessing this resource		4.83 ± 0.41 (5)
I think parents and caregivers will have an increased understanding of opioid use treatment services in their community after accessing this resource		4.67 ± 0.52 (5)
**Final thoughts**		
As a result of my participation in this working group, I feel hopeful about the future of opioid use treatment services for youth in BC	3 ± 0.83 (3)	3.5 ± 0.55 (3.5)
Overall, I am satisfied with the working group	4.88 ± 0.35 (5)	4.33 ± 0.82 (4.5)
This working group is a good use of my time	4.88 ± 0.35 (5)	4.83 ± 0.41 (5)
**Open-ended questions**		
What were the strengths (or best parts) of the working group?		
What could be improved about the working group?		
How has the COVID-19 impacted the co-design process?		
Is there anything else you would like the ITT project team to know about your experience with the working group? *(Was there anything from the previous questions you wanted to elaborate on*? *This is where you can do that*!*)*		

Likert scale responses: Likert scale responses: 1 = Strongly disagree; 2 = Disagree; 3 = Neither agree nor disagree; 4 = Agree; 5 = Strongly agree

SD: standard deviation

^a^: Response rate was 67% (8/12 completed). However, the 4 caregivers who did not respond attended 1–2 meetings before withdrawing from the co-design meetings due to a lack of capacity to remain engaged.

^b^: One participant responded “I don’t know”, which was given the same value as “Neither agree nor disagree”.

^c^: Questions that were only asked in the endpoint survey (after the handbook content was developed).

Caregivers acknowledged that their voices may not be representative of all caregivers’ experiences, given that they mainly identified as white, middle class, and female. This was also captured in the endpoint evaluation survey, where caregivers described missing perspectives from people of color, new immigrants, fathers, grandparents, and foster parents. This was acknowledged as a limitation and included as a disclaimer in the handbook, which encourages others to adapt the handbook to reflect the needs and experiences of caregivers in their community, supported by an adaptation toolkit available upon request.

Strengths of the working group included the ability to share and learn about everyone’s lived experiences, along with resources that caregivers had found helpful throughout their journeys. Although most participants would have preferred to meet in person, the online breakout rooms helped make the meetings more productive virtually. Caregivers also highlighted the impact of good facilitation skills by the co-leads, which resulted in meetings described as well-organized, clear, and well-paced. Some caregivers expressed wanting to spend more time on writing and editing rather than sharing stories, and thus working group members were given opportunities to write content outside of meeting times to support different working styles and increase productivity during meetings.

From the open-ended questions, caregivers reflected that the co-design process made them “*feel not alone*” and acted as a support group in itself: *“I’m very much still in it [my young person’s substance use journey] and I appreciate the therapeutic aspect of this process*.*”* For others, it acted as a source of hope: *“*…*it’s always just great to connect because I’m still feeling very much in the thick of my journey*, *but it just gives me hope that we’re all here*.*”* The process also helped caregivers build resilience by creating something meaningful from their own lived experience and helping other caregivers. As one caregiver described: *“This type of thing gives me strength*. *When we feel helpless for our kids*, *doing something like this really helps take that feeling away*.*”* Another caregiver described the process as *“self-care for me*, *just to be able to put the knowledge that we’ve accumulated through this journey to good use makes me feel good*.*”*

### Case study 2: Co-designing a virtual OAT guide to support youth making opioid use treatment decisions in Kelowna and Vancouver, BC

#### Rationale for prototype selection

This second case study reports the co-design of a youth-friendly virtual OAT guide with community-based partners at Foundry Vancouver-Granville and Foundry Kelowna. This prototype was chosen in response to the communities’ need to educate youth and service providers about OAT, including current and local OAT medication options and important information for those considering OAT as a treatment option. The original idea came from a service provider workshop in Kelowna, BC (Phase 1) to educate service providers about OAT and help them build trusting relationships with youth; however, community partners and youth team members expressed the need for youth friendly information about OAT, therefore both stakeholder groups (youth and service providers) were engaged during the development and implementation of the guide. A collaborative approach was taken with two community-based partners, which allowed youth from two different communities (urban and suburban centers) to share their unique perspectives about OAT services.

#### Co-design team

Three CCs were onboarded to co-lead the local development and implementation of the virtual OAT guide, working closely with an ITT project team lead (author CK) and a youth team member with lived experience of opioid use. In Vancouver, a senior and a junior youth peer supporter were onboarded as CCs. Both peer supporters had existing relationships with youth who were accessing OAT and thus could easily engage with youth in the co-design process. The senior peer supporter also had lived experience with OAT, and therefore played a large role in the content development. In Kelowna, a youth peer supporter was initially hired for the role; however, due to staff turnover, a mental health clinician was onboarded to the position mid-way through the co-design process. This CC did not always attend the co-design sessions but supported the work by connecting the team with colleagues who could facilitate the engagement of youth with OAT experience. The CC also supported communications, approvals, and administration at the center level. Over the course of the development process, a total of 10 youth from Vancouver and Kelowna, who were actively receiving OAT or had received OAT in the past five years, took part in the project. Youth who attended the Phase 1 workshops were invited to participate, while the CCs recruited youth through their networks. A nurse practitioner from the a Phase 1 workshop was also involved in the content development process to ensure the content was informed by clinical evidence and current OAT prescribing best practices. The team’s structure and level of engagement varied over the course of the co-design process. All participating youth and service providers were provided with a $25/hour honorarium for their time.

#### Co-design process

**Fig 3:**
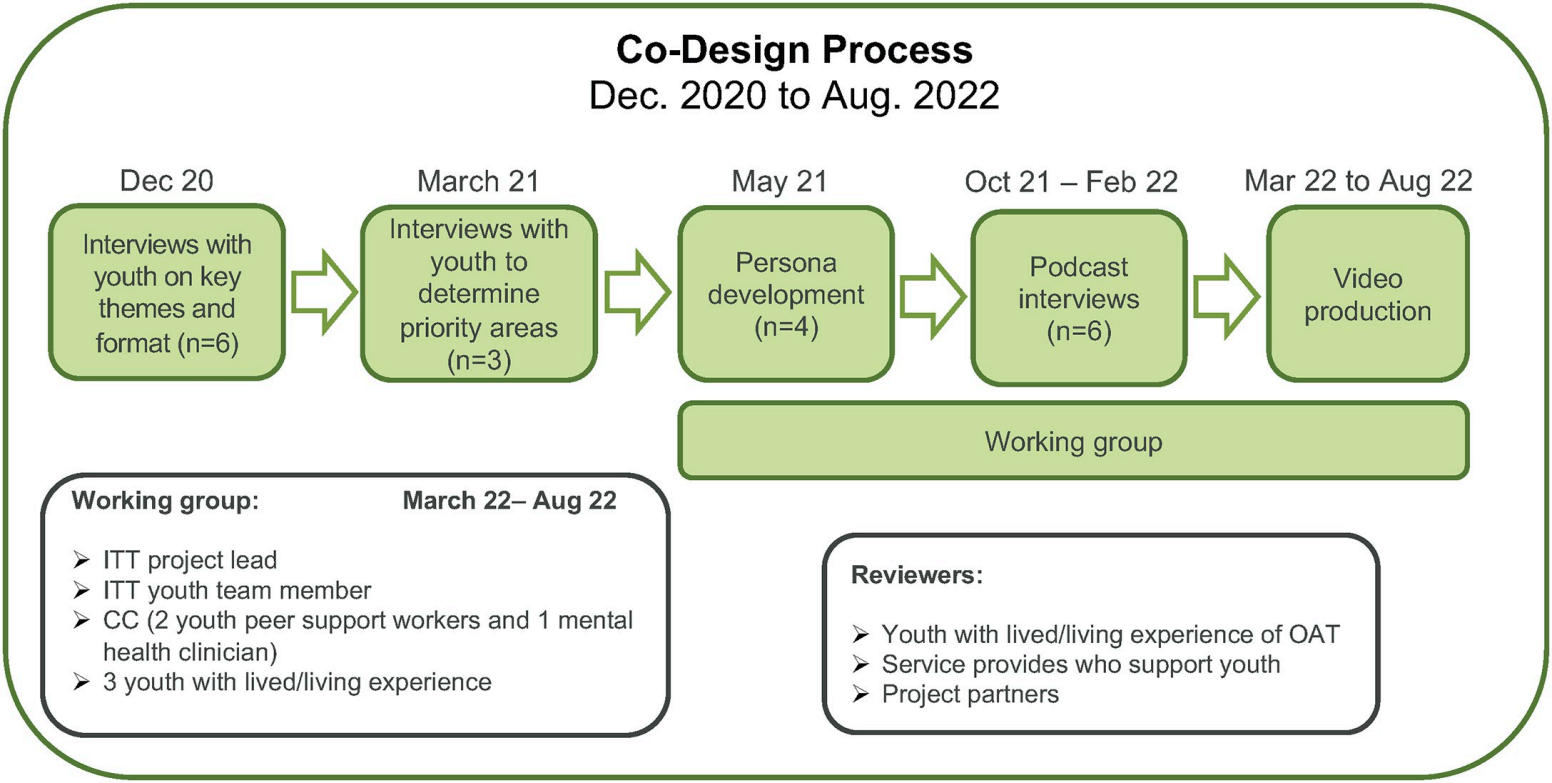
Co-design process for the virtual OAT guide.

The content development for the virtual OAT guide took place December 2020 to March 2022 (see Fig 3). Two rounds of interviews were conducted between December 2020 and March 2021 with six youth who were receiving OAT. CCs led the in-person interviews, with the project team lead supporting virtually. For the first round, we conducted six interviews to identify topics that would be helpful for youth embarking on their OAT journey. This involved asking youth *“What do you wish you had known about OAT that you know now*?*”* and how the content should be delivered to youth (e.g., “*How long should the OAT videos be*?*”*, *“Where should the OAT guide be accessed*?*”*). A second round of interviews was conducted with three youth to prioritize key topics for the virtual OAT guide.

Taking these learnings, the project team lead drafted the initial evidence-based content, which was informed by current literature and then reviewed and validated by a variety of subject matter experts (e.g., nurse practitioners, physicians, a pharmacist, academic researchers). To present information about youths’ experiences with OAT, character personas that captured a “day in the life” of youth on OAT were created through additional consultations with youth. The local CCs determined the mode of consultation in each community. In Vancouver, the CCs hosted an in-person focus group with two youth who completed a journey map activity, while two individual interviews were conducted with youth in Kelowna. This led to the development of two personas: Sam, a 20-year-old man from Vancouver on methadone, and Sasha, an 18-year-old woman from Kelowna on Suboxone^®^.

To design the guide, a vendor specializing in inclusive facilitation and youth-friendly video production was onboarded in September 2021. The original idea for the guide involved a virtual reality tour of what it would be like to access OAT; however, this was not possible due to unpredictable public health restrictions during the COVID-19 pandemic. Through a creative brainstorming session, the ITT project lead, CCs, and two youth with living experience of OAT decided to create an animated video series using a podcast format, which would involve a podcast host interviewing youth from Kelowna and Vancouver about their experiences with OAT. As a result, the project team completed six additional podcast interviews (five with youth with living experience and one with a nurse practitioner) between October 2021 and February 2022 and incorporated their stories into the videos. The CCs joined the youth in-person while the youth were interviewed and recorded virtually by the vendor and the project team lead. Youth were asked to share their stories and experiences with OAT (e.g., what a typical day looks like for them, the positive and negative aspects of their current OAT regimen) and give advice to other youth who may be considering OAT. The nurse practitioner was asked to describe OAT, where youth can access it, and why it is important. The interview audio was used to complement and authenticate the journeys of Sam and Sasha (the personas). A youth artist based in Vancouver was also hired to create the artwork for the videos, in collaboration with the video production team.

A working group was formed to revise the OAT guide’s content, determine the structure and flow for the videos and audio, review the artwork, and inform the implementation. This group met bi-weekly over Zoom between March 2022 and August 2022 and involved the project team lead, youth interviewees, and the CCs from Vancouver and Kelowna. These meetings also gave the interviewees an opportunity to review their own podcast transcripts to ensure they were comfortable with what would be shared in the videos. A community agreement was co-created and used as a grounding tool at the start of each meeting. Other safety considerations included regularly checking-in with other working group members and using communication tools such as texting and private Zoom chats.

The scripts and artwork for the OAT guide went through multiple rounds of revisions by the working group. Five subject matter experts revised the clinical content from the scripts and videos to ensure that the information was current and evidence-informed. The OAT guide was also reviewed by leadership teams from all partnering organizations (Foundry Vancouver-Granville, Foundry Kelowna, Foundry Central Office, and CCSA). Youth ultimately had final say on any decisions made. We were unable to test the videos with other youth in the community outside of the co-design process due to restrictions in project budget and timelines. Restrictions were compounded by challenges engaging youth over the course of the COVID-19 pandemic and the complex feedback cycle among the working group, the four partnering organizations, and the video production team.

Youth highlighted the importance of making the OAT guide accessible and sharing it from a reliable organization for credibility. As a result, the finalized OAT guide, comprising four unique episodes (or videos), was posted on the Foundry YouTube channel (@foundrybc2819). We also designed an OAT infographic that describes each OAT option and its pros and cons, according to youth, to complement the videos, in addition to a resource list of community services in Vancouver and Kelowna to support continuity of care.

#### Co-design process evaluation

Anonymous midpoint and endpoint surveys were distributed to evaluate the co-design process. The midpoint survey was distributed to the initial six interviewees, working group members, and content experts in March 2022 (once a formal working group had been established), while the endpoint survey was solely distributed to the working group members in August 2022 (when the animated videos were finalized). As with the caregiver handbook, surveys were adapted from PPEET [56] and used Likert-type and open-ended questions, which were completed in Qualtrics^®^ and did not collect any personally identifiable information.

The survey questions and responses can be found in Table 3. Seven people (three interviewees and four working group members) completed the midpoint survey, while four working group members filled out the endpoint survey. Overall, youth, working group members, and/or content experts described feeling adequately supported to participate in the project and felt that participating was a good use of their time. As one participant described: “*I feel very fortunate to have been involved in this project from the beginning and to see how it has evolved*! *It is a great learning opportunity and inside look at how resource/educational tools are created from inception to finished product with youth health at the forefront*.”

**Table 3:** Virtual OAT guide midpoint and endpoint process evaluation survey results.

Midpoint and Endpoint Survey Questions	Midpoint survey (n = 7) ^a^	Endpoint survey (n = 4) ^b^
	Mean ± SD (Median)	Mean ± SD (Median)
**Part A: Communication and support for participation**		
I have a clear understanding of the purpose of my role as an interviewee/project team member	4.86 ± 0.38 (5)	5 ± 0 (5)
The supports I need to participate are available (i.e., information, compensation, access to technology, etc.)	4.57 ± 0.53 (5)	4.75 ± 0.5 (5)
The COVID-19 pandemic made it more challenging for me to participate in the interview/project	2.86 ± 0.90 (3)	2.75 ± 0.96 (2.5)
I have enough information to contribute to the topic being discussed	4.71 ± 0.49 (5)	4.25 ± 0.5 (4)
The interview(s)/meeting(s) were scheduled at a time that was convenient for me	4.57 ± 0.53 (5)	4.5 ± 0.58 (4.5)
My role and responsibilities were clearly explained to me before the interview/project began	4.57 ± 0.79 (5)	5 ± 0 (5)
My role met my expectations	4.29 ± 0.76 (4)	4.25 ± 0.5 (4)
I have learned valuable information/skills through this process	4.75 ± 0.5 (5)	4.5 ± 1 (5)
**Part B: Sharing your views and perspectives**		
I am able to express my views freely	4.75 ± 0.5 (5)	4.5 ± 1 (5)
I feel that my views are heard	4.75 ± 0.5 (5)	4.75 ± 0.5 (5)
Overall, I was satisfied with the ITT interview/ITT working group	4.71 ± 0.49 (5)	4.5 ± 0.58 (4.5)
I felt the interview/working group was a good use of my time	4.86 ± 0.38 (5)	4.75 ± 0.5 (5)
**Part C: Impact and influence of the work**		
I am confident the input I provided will be used by the ITT project team and their project partners (i.e., Foundry and CCSA)	4.25 ± 0.5 (4)	4.5 ± 0.58 (4.5)
I think that the Virtual OAT Guide responds to the needs identified by members of the community	4.25 ± 0.5 (4)	4 ± 0 (4)
I think the Virtual OAT Guide will be useful to youth considering accessing OAT	4.71 ± 0.49 (5)	4.5 ± 0.58 (4.5)
I think youth will be satisfied with the Virtual OAT Guide	4.14 ± 0.38 (4)	4.5 ± 0.58 (4.5)
I would share the Virtual OAT Guide with other youth	5 ± 0 (5)	5 ± 0 (5)
I think youth will have an increased understanding of OAT after watching this resource	4.43 ± 0.53 (4)	4.25 ± 0.5 (4)
**Final thoughts**		
As a result of participating in the ITT project, I feel hopeful about the future of opioid use treatment services for youth in BC	4.14 ± 0.69 (4)	4.25 ± 0.5 (4)
**Open-ended questions**		
Who do you think will benefit from the virtual OAT guide?		
What are some benefits you think the virtual guide will have for other youth using opioids?		
What were the strengths (or best parts) of this process?		
What could be improved about this process?		
How has the COVID-19 pandemic impacted this process?		
Is there anything else you would like the ITT project team to know about your experience with the working group? *(Was there anything from the previous questions you wanted to elaborate on*? *This is where you can do that*!*)*		

Likert scale responses: Likert scale responses: 1 = Strongly disagree; 2 = Disagree; 3 = Neither agree nor disagree; 4 = Agree; 5 = Strongly agree

SD: standard deviation

^a^: Response rate was 58% (7/12 completed). This survey was sent to the working group members, youth who took part in an interview, and content experts.

^b^: Response rate was 67% (4/6 completed). The survey was sent to the working group members.

Working group members agreed that the chosen prototype was reflective of the needs of their community, and all respondents anticipated that the virtual guide would have a positive impact on youth. When asked about the potential benefits of the virtual OAT guide, respondents mentioned that the guide would *“show [youth] there is hope and that they’re not alone”*, *“give [youth] a better sense of agency over their OAT process and deciding what is right for them”*, and *“normalize and humanize the experience of substance use struggles and OAT”*. Another respondent described: *“I really feel like these videos will help youth understand OAT more and maybe even give it a chance*.*”*

When asked about the strengths of the co-design process, working group members shared that they appreciated the opportunity to share their stories and co-create with others, and they valued *“being connected to our team members and being heard”*. Interviewees also appreciated being given the opportunity to share their voice on a topic that was important to them. Other benefits included sharing information and brainstorming with both youth and service providers and having a *“strong representation of youth perspectives/experiences to inform the project”*. Reported weaknesses included a limited ability to connect in person due to the COVID-19 pandemic and the length of the co-design process, which impacted the opportunity to engage with more youth.

## Discussion

The case studies presented here provide an overview of how CBPR and co-design methods can be used together to develop innovations to support youth, caregivers, and service providers when accessing or providing youth opioid use treatments/services. Using CBPR methods allowed us to establish strong community partnerships to inform and support the project’s activities across all phases, such as organizing and conducting the community- and stakeholder specific workshops (Phase 1); selecting relevant prototypes to address the needs of each community and identifying and supporting individuals to co-design innovations (Phase 2); and supporting local implementation and evaluation (Phase 3). Meanwhile, co-design methods provided creative ways to engage with those receiving or providing services to better understand their needs and experiences and design innovative solutions (Phase 1); co-design community-specific innovations based on the Phase 1 findings and stakeholders expertise (e.g., lived experiences) (Phase 2); and co-develop implementation and evaluation strategies (Phase 3).

During Phase 2, community partners provided important perspectives to select prototypes that would best meet the needs of their community, while also considering available resources to ensure the project’s success. Unique resource considerations included potential staff to take on the CC position and existing relationships and channels to support engagement. These relationships became more important as we faced ongoing challenges and restrictions due to the COVID-19 pandemic, which impacted our ability to recruit and engage relevant stakeholders. As such, having CCs who worked directly with youth, caregivers, and other service providers helped us identify co-design partners. Their specific role as peer supporters also enhanced our ability to create safe spaces for youth and caregivers, build trust, and mitigate power dynamics among youth, caregivers, and the ITT project team members.

Establishing trusting relationships is an important success factor for patient and family engagement, CBPR, and co-design [57–60]. Project objectives, engagement strategies, and overall scope of co-design projects tend emerge over time through community engagement, therefore participants may have difficulties understanding what full participation entails when first approached. Timing is also an important factor when engaging patients and families who have competing commitments, which can include managing health conditions and recovery [60, 61]. Consequently, working collaboratively with service providers and healthcare organizations using CBPR can help recruit participants in the early stages of co-design and support ongoing engagement while navigating competing priorities. For example, Heslop and colleagues (2022) [15] appointed a clinical champion to enhance clinician and patient engagement in the co-design process and support the overall success of a project. Similarly, working with CCs who committed much of their time, skills, and efforts to this project was invaluable to the co-design process.

Including trusted CCs on the team also helped us select appropriate engagement methods for co-design. While a structured working group of caregivers was engaged throughout the entire development of the caregiver handbook, we continuously adapted our engagement methods when designing the OAT guide due to shifting engagement preferences among participating youth and service providers, as well as shifts in project goals (e.g., recording podcast material, developing personas, reviewing artwork). Co-design projects often require creative engagement strategies that are tailored to stakeholders’ availability and local context [58, 60, 61]. For instance, Girling and colleagues (2022) [62] reported significant challenges recruiting youth from community forensic settings to understand their service experiences and co-design service improvements. This led them to attempt several different recruitment strategies and to use observational data when youth were unable to participate. Although flexibility is an important aspect of co-design, efforts should be made to engage service users as equal decision makers whenever possible [63]. One of the strengths of our study was the level of youth and family involvement in the co-design, production, and implementation of each innovation. This was supported by involving youth and caregivers with lived experience in each working group, where major decisions about the prototypes and final innovations were made.

Power dynamics among patients, families, and service providers are also important to consider when planning co-design studies [63]. Service users and service providers are often concurrently engaged in co-design to bring multiple perspectives together and increase service providers’ understanding of service user experiences [10, 12, 15, 16, 60]. Yet, this can lead to conflicting priorities among stakeholder groups and result in negative co-design experiences if service user perspectives are not properly considered [12, 15, 17, 35]. People who use substances can be subjected to stigma and negative attitudes from service providers, which can undermine their autonomy, self-confidence, and self-worth [35, 41, 51]. Youth are often left out of treatment decisions and lack meaningful engagement when designing health interventions and policies [35, 41]. As such, in our work, service users (i.e., youth, caregivers) and service providers were engaged separately. Youth and caregivers were also given more decision-making power as they were the primary intended audience for each innovation.

When working with people with lived experience of substance use, a trauma-informed approach is recommended to foster safe spaces and avoid re-traumatization [41, 64]. Although it was impossible to remove all risks given that the topics discussed were difficult and triggering by nature (e.g., overdose, negative service experiences, side effects of OAT), efforts were made to minimize these risks by sending meeting agendas in advance, co-creating a community agreement, and giving youth and caregivers the flexibility to choose how to engage with the project and take a step back when needed. Having recruited participants through existing relationships with peer supporters (who were also the CCs) created a greater sense of safety for participants to share their stories and manage difficult situations as they arose. Tragically, two youth opioid-related deaths occurred over the course of the project, which impacted caregiver partners. Having a CC who was already a source of support for caregivers helped us navigate these difficult situations and resulted in a two-month meeting break, during which the project team shifted focus to other aspects of the co-design process (i.e., research, writing, consulting with content experts).

The two case studies demonstrate how integrating CBPR and co-design methods can facilitate patient, family, and service provider engagement in the development of innovative solutions and how this process can benefit those participating in the co-design process. Although there is growing evidence demonstrating the acceptability of co-designed interventions [11–17], few studies have evaluated the process of co-design and its impact on partners. Those that have report the importance of good facilitation to introduce knowledge and tools to support constructive conversations [59], as well as frequent and ongoing communication with co-design partners [12, 59]. Ramos and colleagues (2020) [59] also reported how patients appreciated being able to give something back to improve other patients’ experiences, which is consistent with what we heard from youth, caregivers, and service providers in this study. This study demonstrates how CBPR and co-design can result in innovations that are highly accepted by those involved in the design process.

The developed innovations directly responded to the needs of youth (virtual OAT guide) and caregivers (caregiver handbook), which were expressed during the Phase 1 workshops [24, 53, 54] and supported by the literature. For example, although OAT is a first-line evidence-based intervention to treat OUD among youth and adults, youth have described being dissuaded by family members and service providers about accessing OAT or being denied certain OAT options (e.g., full opioid agonists such as methadone) [31, 33]. As such, the virtual OAT guide provides unbiased information about OAT to empower youth to make their own treatment decisions and support conversations between youth and service providers about treatment options. However, further implementation research will be important to assess and understand the real impact of each innovation.

### Limitations

CBPR and co-design processes are often lengthy and laborious [15–17], which can limit their applicability in resource-constrained settings, especially when mitigating tight funding requirements and emerging public health priorities (e.g., COVID-19, drug toxicity and overdose crisis). Although we were able to secure funding extensions to support the equitable involvement of co-design partners, conducting these activities within reasonable timelines was important for our partners and the team’s capacity. We therefore relied on existing networks of youth, caregivers, and service providers to take part in the co-design and the review processes. Though leaning on existing networks was beneficial to mitigate delays in project timelines, participants expressed that there were other voices in their community that warranted engagement. Sampling bias is a common phenomenon in co-design, given the time commitment expected by partners and disease burden [60, 61]. This can lead to the exclusion of certain populations, including individuals who have limited support available to help manage health concerns, those with less education, and those with limited computer literacy skills. This highlights some of the shortcomings of co-design and the need to make active efforts to engage end-users with diverse experiences and perspectives. Although we engaged a diverse group of youth, caregivers, and service providers (n = 80) in the Phase 1 workshops to overcome these limitations, the obstacles we faced due to the COVID-19 pandemic (e.g., unpredictable public health restrictions) made this challenging in Phase 2.

In an effort to reach more diverse voices in Phase 2, we tested the caregiver handbook with service providers and caregivers from across the province to validate the resource with other populations and settings before wider implementation. This came with its own limitations as feedback was provided through numerous methods (e.g., email responses, direct comments throughout the handbook’s content) rather than through the feedback form. Additional efforts to increase completion of the feedback form may have increased our ability to collect consistent data, such as distributing these forms via a survey link with an attached honorarium. Meanwhile, we were unable to test the OAT guide with other populations due to engagement challenges and project timelines. While traditional co-design methods involve continuous cycles of testing and redesign [11, 39], which can facilitate more widespread engagement, this proved challenging given that both innovations focused on storytelling and experiential knowledge. Thus, feedback mainly centered around the format, design, and highlighting what resources, information, or perspectives may be lacking. While this may have limited the generalizability of the co-designed solutions, their purpose was not to make one-size-fits-all recommendations, but to highlight how everyone’s journey is different, reduce stigma, and encourage youth and caregivers to find a path that works for them. Implementation research will be important to assess the resources’ applicability for youth, caregivers, and service providers across broader community contexts.

## Conclusion

This study demonstrates the benefits of weaving CBPR and co-design to develop innovative solutions aimed at improving the healthcare experiences of underserved populations, such as youth who use opioids and their families. Given the time and resource intensive nature of co-design, integrating CBPR can help facilitate strong partnerships that are integral to its success. Using these complementary methods can strengthen the ability to recruit, support, and engage service users and service providers in co-design, create flexible pathways for engagement, and ensure solutions are endorsed and accepted by service users, service providers, and communities.
